# Remote and Anesthetic-Induced Myocardial Preconditioning Is Preserved in Atherosclerotic LDL Receptor-/- Mice In Vivo

**DOI:** 10.1155/2021/5596590

**Published:** 2021-05-24

**Authors:** Walter Petermichl, Kathrin Eglmeier, Henriette Hesse, Michael Gruber, Bernhard Graf, Andre Bredthauer, Andreas Redel

**Affiliations:** ^1^Department of Anesthesiology, University of Regensburg, Franz-Josef-Strauß-Allee 11, 93053 Regensburg, Germany; ^2^Department of Anesthesiology, Amberg Hospital, Mariahilfbergweg 7, 92224 Amberg, Germany

## Abstract

**Introduction:**

In the animal model, preconditioning is a powerful weapon against ischemic damage. The reason why the human heart cannot be protected from ischemic damage by preconditioning remains unclear. There are assumptions that the lack of preconditioning in humans is caused by concomitant diseases such as dyslipoproteinemia and arteriosclerosis. This study investigates whether dyslipoproteinemia and the resulting arteriosclerosis can be a cause of a reduced precondition effect of heart in mice.

**Methods:**

LDL receptor-deficient mice were fed a long-term (14-16 weeks) high-fat atherogenic diet to induce arteriosclerosis. Arteriosclerosis was identified by histological examination and vessel contraction tests. LDLR-/- and wild-type mice were randomly assigned to anesthetic-induced, remote ischemic, or no preconditioning. All mice were subjected to 45 minutes of coronary artery occlusion and 180 minutes of reperfusion. The area at risk and infarct size were determined by Evans Blue and triphenyltetrazolium chloride staining.

**Results:**

Histopathological examination showed atherosclerosis in high-fat atherogenic fed LDLR-/- mice, and the vessel relaxation capacity was significantly reduced compared to wild-type mice. In the wild type, as expected, infarct size was significantly reduced by preconditioning compared to the control. In LDLR-/- mice, infarct size was significantly reduced by preconditioning compared to the control. Surprisingly, the LDLR-/- control group also had a significantly reduced infarct size compared to the wild-type control group.

**Conclusion:**

We were able to demonstrate that a high-fat diet morphologically and functionally triggered atherosclerosis in LDLR-/- mice. Interestingly, LDLR-/- mice with an atherogenic diet had smaller infarct sizes compared to wild-type mice. Moreover, preconditioning additionally reduced myocardial infarct size in LDLR-/- mice. A long-term high-fat atherogenic diet and preconditioning seem to result in additive cardioprotection in LDLR-/- mice.

## 1. Introduction

Acute myocardial infarction (AMI) is one of the leading causes of mortality and morbidity worldwide. There has been an extensive search for cardioprotective therapies to reduce myocardial ischemia-reperfusion (IR) injury. Ischemic preconditioning is the intriguing phenomenon whereby brief and transient episodes of ischemia that, as such, do not cause tissue necrosis instead render tissue and organs resistant to a sustained ischemic insult. Since the first report by Murry et al. of preconditioning to prevent ischemia reperfusion injury (IRI), numerous studies have been conducted [1]. In the following years, many in vitro and in vivo studies have provided strong evidence for the infarct-sparing potential of preconditioning, be it ischemic (IPC), remote ischemic (RIPC), or anesthetic-induced (APC) preconditioning. The overall effect of preconditioning in animal studies has raised hopes for finding an infarct-sparing intervention in the clinical setting. However, data from large randomized prospective studies have been disappointing [2].

IPC with repeat episodes of brief ischemia within one vascular bed protects the organ (e.g., myocardium) from a subsequent prolonged episode of ischemia in that same vascular bed [3]. First described by Murray, this IPC technique is now used predominantly in the experimental setting. In RIPC, the ischemic stimulus is applied in an organ remote to the target organ. For example, transient ischemia in the lower limb, prior to coronary artery occlusion, reduced infarct size in a rabbit model [4]. Neuronal and humoral components are involved in the signal transfer from the peripheral stimulus site to the target organ, serving as protective mediators [5]. These mediators can also be triggered by anesthetics in the absence of preconditioning ischemic periods [6]. Several conditions might account for the mitigated preconditioning effect in clinical studies. A number of factors have been shown to mitigate preconditioning, including advanced age and being of female sex, as well as hyperglycemia and *β*-adrenergic antagonists [7–10]. Moreover, it is suggested that atherosclerosis with concomitant endothelial dysfunction inhibits preconditioning. This study's authors have demonstrated previously that anesthetic-induced preconditioning is absent in eNOS knockout mice with endothelial dysfunction [11]. Reports of preconditioning in atherosclerotic animals are inconsistent. In rabbits and rats, hyperlipidemia and arteriosclerosis have been shown to abolish preconditioning [12, 13]. However, other studies report successful IPC or RIPC in hyperlipidemic and atherosclerotic animals [14]. In isolated hearts of atherosclerotic mice, preconditioning has been reported to be effective [15]. Whether atherosclerosis abolishes preconditioning in vivo in mice has not been investigated. Therefore, we induced atherosclerosis through a high-fat diet in LDLR-/- mice and subjected these animals in vivo to myocardial infarction with or without anesthetic-induced and remote ischemic preconditioning.

## 2. Material and Methods

### 2.1. Animals

All experimental procedures and protocols used in this study were reviewed and approved by the government of Lower Franconia's (Bavaria, Germany) animal care and use committee (54-2532.1-18/14). All experiments conformed to the American Physiological Society's guiding principles in the care and use of animals and were in accordance with the guide for the care and use of laboratory animals [16].

Eight-week-old male C57BL/6 (WT) mice and LDLR-/- (background C57BL/6) (LDLR-/-) mice were used for all experiments (Charles River Laboratories, Sulzfeld, Germany). The animals were housed under controlled conditions (22°C, 55-65% humidity, 12 h light-dark cycle) and were allowed free access to tap water. After a one-week acclimatization, LDLR-/- mice were fed a high-fat diet (SM M-Z, atherogenic, +1% cholesterol, Basis V1124) (ssniff Spezialdiäten GmbH, Soest, Germany) for 14–16 weeks. Wild-type mice were fed a standard diet. All mice were subjected to experiments between 22 and 26 weeks of age.

### 2.2. Surgical Procedure

Pentobarbital sodium was used for anesthesia induction (60 mg/kg body weight i.p.) and maintenance (10 mg/kg/h i.p.). The mice were placed on a temperature-controlled heated table (RT, Effenberg, Munich, Germany) with a rectal thermometer probe attached to a thermal feedback controller to maintain body temperature at 36.5°C–37.5°C. After anesthesia induction, the mice were secured in a supine position with the upper and lower extremities attached to the table with a removable tape. The trachea was surgically exposed, and a blunt polyethylene cannula (Insyte 22 g, Beckton Dickinson) was inserted into the trachea via tracheotomy. All mice were mechanically ventilated with a 50 : 50 air/oxygen mixture using a rodent ventilator (SAR 830/AP, CWE Inc., Ardmore, PA, USA). A 3-lead needle-probe electrocardiogram (ECG) was attached to the right common carotid artery, and a saline filled PE-10 catheter was inserted into the right jugular vein. The hemodynamic parameters, namely, heart rate (HR), mean arterial pressure (MAP), and temperature, were measured at the end of the memory phase (MEM15′), at the end of the coronary artery occlusion (CAO), and 60 minutes after reperfusion (REP60′).

**Figure 1:**
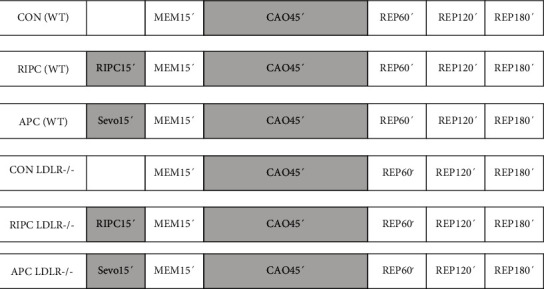
Study protocol and time line. CON WT: control group (C57/BL6 wild type) no preconditioning; MEM15′: memory phase over 15 minutes; CAO45′: coronary artery occlusion over 45 minutes; REP60′, 120′, and 180′: reperfusion phase 60, 120, and 180 minutes after COA45′; RIPC WT: remote ischemic preconditioning group (C57/BL6 WT); APC WT: anesthetic-induced preconditioning group (C57/BL6 wild type); CON LDLR-/-: control group (C57/BL6 LDL-receptor deficiency); RIPC LDLR-/-: remote ischemic preconditioning group (C57/BL6 LDL-receptor deficiency); APC LDLR-/-: anesthetic induce preconditioning group (C57/BL6 LDL-receptor deficiency). At the end of REP180′, the mouse was sacrificed and the heart was isolated.

The mice were randomly assigned to 6 groups (*N* = 8 per group). Control group mice received no further treatment in the time-matched period prior to CAO. APC group mice were preconditioned with 1.0 MAC sevoflurane (Sevorane®, Abbott, Wiesbaden, Germany; 3.4 Vol-%) prior to CAO [17]. Sevoflurane was administered for 15 minutes via a respirator 30 minutes prior to CAO [18]. RIPC group mice were subjected to a 15-minute occlusion of the left lower limb arteria before coronary artery occlusion using a tourniquet. All protocols were performed in both WT and LDLR-/- mice. Anesthetic-induced and remote ischemic preconditionings were followed by a memory phase of 15 minutes prior to COA. This experimental protocol is illustrated in Figure 1.

In all groups, coronary artery occlusion was performed under an upright dissecting microscope (Zeiss Olympus SZX12, Jena, Germany). After a left anterior thoracotomy, exposure of the heart, and dissection of the pericardium, the left coronary artery (LCA) was visually identified. The LCA was ligated at the site of its emergence from under the left atrium, resulting in a large myocardial infarction involving the anterolateral, posterior, and apical regions of the heart [18]. Once visually identified, an 8-0 nylon suture (Prolene, Ethicon, Norderstedt, Germany) was placed around the LCA. CAO was performed using a hanging-weight system [19]: in short, the LCA suture was threaded through a small piece of blunt-edged plastic tube (PE-10 tubing), and two small weights (1 g) were attached to each end. When the weights hung freely, the LCA was immediately occluded. LCA occlusion terminated once the weights were supported. Successful LCA occlusion was confirmed by an immediate change in the color of the vessel (from light red to dark violet), the change in color of the myocardium supplied by the vessel (from bright red to white), and the presence of ST elevations in the ECG. During reperfusion, the changes in color instantly disappeared when the hanging weights were supported and the LCA was reperfused. During the entire procedure, the heart was kept wet with a humid piece of absorbent cotton.

### 2.3. Measurement of Area at Risk and Myocardial Infarct Size

Myocardial infarct size (IS) and area at risk (AAR) were determined as described by Redel et al. [18]: after reperfusion, the LAD was reoccluded and 1 mL Evans Blue (0.1 g/mL; Sigma-Aldrich, Taufkirchen, Germany) and was slowly injected into the carotid artery to delineate the AAR. After a lethal dose injection of pentobarbital (150 *μ*g/g i.p.), the heart was rapidly removed. The left ventricle was dissected and cut into 8 transverse slices 1 mm thick using an acrylic heart matrix (Aster Industries, McCandles, PA, USA). All slices were incubated with 2% triphenyltetrazolium chloride (TTC, Sigma-Aldrich, Taufkirchen, Germany), fixed overnight in 10% formalin, weighed, and digitally photographed (Finepix S3 Pro, Fujifilm, Tokyo, Japan). These photographs were then analyzed with picture analysis software (Adobe Photoshop CS 8.0.1; Adobe Systems Inc., San Jose, CA, USA) and the infarcted area (IA), AAR, and normal zone (NZ) were quantified by an investigator blinded to the treatment protocol. The resulting fractions of IA, AAR, and NZ of each slice were multiplied by the weight of the slice. Infarct size was calculated by the following formula: infarct size = weight of infarcted area/weight of area at risk × 100 (IS = IA/AAR × 100). Mice with an AAR of less than 20% of the left ventricle were excluded from further analysis.

### 2.4. Histological Analysis of the Aortic Root

To verify atherosclerosis in high-fat/atherogenic fed LDLR-/- mice, hematoxylin-eosin- (HE-) stained sections of the aorta were qualitatively examined and compared with those of wild-type mice. The aortic root was isolated, embedded in Tissue-Tek (T.O.C. compound, Sakura Fine Technical, Tokyo, Japan), and snap frozen in liquid nitrogen. OCT-embedded samples were cut serially into cross-sections (5 *μ*m thick), and hematoxylin-eosin staining was carried out according to the manufacturer's instructions. Using a microscope (Leica, Wetzlar, Germany, 40x magnification), typical arteriosclerotic morphology (foam cells, intima thickness, and fibrotic cap) was quantitatively analyzed.

### 2.5. Vessel Contraction Myography

After euthanizing the mouse, the vascular system was perfused and the mesenteric vascular system was dissected. Two branches of the superior mesenteric artery were removed for experimental purposes. These vessel segments were clamped to a myograph between two capillaries (111P, DMT, Aarhus, Denmark) and perfused with oxygenated Krebs Ringer solution (37°C; pH = 7.4). The lumen and the wall thickness of the vessel were measured with a calibrated video system at three different locations. After an equilibration time, the concentrations of noradrenaline (NA) or acetylcholine (Ach) were raised from 10^−9^ mol/L to 10^−4^ mol/L. Each step introducing a new concentration was separated by a 40-minute wash-out phase with Krebs solution. Next, an irreversible NO synthase inhibitor L-NAME (20-minute incubation time) was administered at a concentration of 10^−5^ mol/L. After a precontraction with 10^−5^ mol/L noradrenaline, acetylcholine was administered in increasing concentrations (10^−10^ to 10^−4^ mol/L), and at each concentration, the dilatation of the vessel lumen was measured.

### 2.6. Statistics

Data are presented as mean ± SEM and were statistically analyzed using the Mann–Whitney test and Kruskal-Wallis test. IBM SPSS Statistics 25 software (SPSS Inc., Armonk, NY, USA) was used for statistical analysis. *p* values of <0.05 were considered statistically significant.

## 3. Results

Of the 67 mice assigned to the experimental protocols, 19 were excluded from the study due to asystole before the examination ended or because of technical problems. Twenty-four additional mice were used for histologic HE-staining of the arteria and a myographic vessel contraction test. A total of 91 mice were used in this investigation.

### 3.1. HE Staining of the Aortic Root

**Figure 2:**
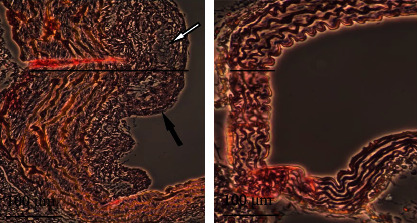
Atherosclerotic vs. normal aortic root. Cross-section and HE staining of mouse aortic root (40-fold magnification). (a) Aortic root of a C57/BL 6 LDLR-/- mouse after 14 weeks of high-fat/atherogenic diet. (b) Aortic root of a C57/BL 6 WT mouse without a high-fat diet. (a) Typical criteria for severe arteriosclerosis can be seen. Significant increase of the aortic wall thickness (black line) compared to the WT aorta in (b). Also further typical atherosclerotic morphological changes can be identified: plaque cap (black arrow), foam cells, and cholesterol crystals (white arrow).

HE staining revealed that in wild-type mice fed a normal diet, the vascular wall was obviously stratified, the intima was smooth and not thickened, there was no lipid deposition under the endothelial cells, and smooth muscle cells in the media were arranged in an orderly manner. In LDLR-/- mice fed a high-fat diet for 12 weeks, there was significant plaque buildup under the intima. The plaque contained foam cells and deposits of cholesterol crystals, the intima was significantly thickened, and the smooth muscle cells in the tunica media were disordered. (Figure 2). This arteriosclerotic metamorphosis was present in all LDLR-/- mice but in none of the wild-type mice.

### 3.2. Body Weight, Temperature, and Hemodynamic Parameters

There was no significant difference in the body temperature of the mice. The body weight of the high-fat diet-fed LDLR-/- mice in the control group was significantly higher compared to that of WT mice (31.0 ± 1.1 g vs. 25.0 ± 0.6 g, *p* = 0.01). In both the APC and RICP groups, the LDLR-/- mice fed an atherogenic diet also had a significantly higher body weight (Table 1).

The heart rate was significantly higher, and the MAP was significantly lower in WT mice compared to LDLR-/- mice in both the control and APC groups during the entire experimental protocol (Table 2).

In the RIPC group, only the MEM15′ MAP was significantly lower in WT mice compared to LDLR-/- mice (55 ± 4 mmHg vs. 63 ± 7 mmHg, *p* = 0.002). There was no significant difference in the other hemodynamic parameters of this group (Table 2).

### 3.3. Area at Risk (AAR) and Infarct Size (IS)

**Figure 3:**
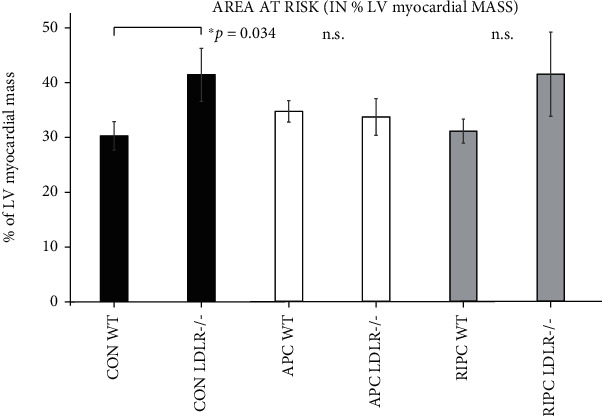
Area at risk in % of the left ventricular area of WT and LDLR-/- mice. Although there is a tendency that LDLR-/- mice have a higher AAR, a significant difference could only be shown in the control group.

There was no significant difference in the area at risk in the intragroup comparison tests of the WT and LDLR-/- groups (Figure 3). The LDLR-/- groups tended to have a larger AAR than the WT mice, though in paired comparisons of the CON, APC, and RIPC groups, only CON LDLR-/- mice had a significantly larger AAR compared to the CON WT (41.4 ± 4.9% vs. 30.3 ± 2.6%, *p* = 0.03).

**Figure 4:**
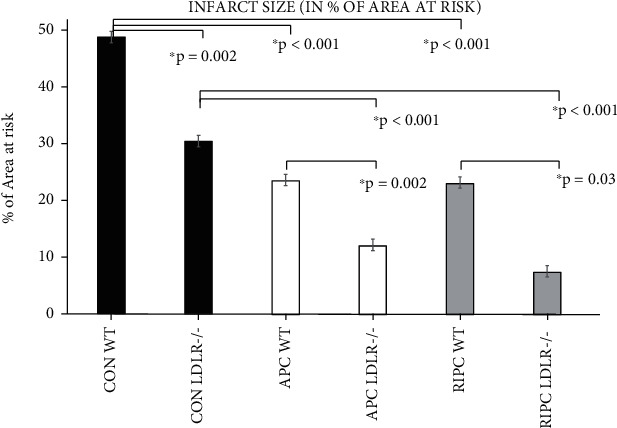
Infarct size: relative IS (IS/AAR) in CON, APC, and RIPC with and without LDLR-/- and high fat diet.

Infarct size was significantly reduced in LDLR-/- mice compared to WT mice (CON LDLR-/- (30.4 ± 4.7%) vs. CON WT (48.0 ± 2.0%), *p* = 0.002; APC LDL-/- (12.1 ± 1.7%) vs. APC WT (23.6 ± 3.8%), *p* = 0.002; and RICP LDLR-/- (7.5 ± 2.7%) vs. RIPC WT (23.1 ± 6.0%), *p* < 0.03) (Figure 4). In wild-type mice, anesthetic-induced preconditioning (23.6 ± 3.8%; *p* < 0.001 vs. WT CON) and remote ischemic preconditioning (23.1 ± 6.0%; *p* < 0.001 vs. WT CON) significantly reduced infarct size compared to the control group (30.4 ± 4.7%). In LDLR-/- mice, anesthetic-induced preconditioning (12.1 ± 1.7%; *p* = 0.005 vs. LDLR-/- CON) and remote ischemic preconditioning (7.5 ± 2.7%; *p* < 0.001 vs. LDLR-/- CON) significantly reduced infarct size compared to the control LDLR-/- mice (Figure 4).

### 3.4. Vessel Contraction Myography

The relaxation capacity of the AMS was significantly reduced in LDLR-/- mice compared to WT mice (72.9 ± 0.9% vs. 89.1 ± 0.7%; *p* = 0.002). The irreversible eNOS inhibitor L-NAME reduced the relaxation capacity of the AMS in LDLR-/- mice from 72.9 ± 0.9% to 60.2 ± 2.7% (*p* = 0.002) and in WT mice from 89.1 ± 0.7% to 74.7 ± 1.5% (*p* = 0.002).

## 4. Discussion

The primary therapy of myocardial infarction (MI) is the immediate revascularization of the occluded vessel. However, the sudden reperfusion of a vessel is not without risk to the ischemic tissue. After primary tissue damage from ischemia, the subsequent reperfusion results in further tissue damage [20]. This effect is known as ischemic reperfusion injury (IRI). Data suggest that free radicals and inflammatory reaction may cause this IRI effect [21, 22]. Preconditioning through repeat sublethal ischemic periods or by pharmacological agents can render cells more resistant to IRI [1]. Numerous studies have been conducted in the past three decades to elucidate the underlying mechanisms and clinical applicability of preconditioning [23]; this treatment induces transcriptional and translational cellular modifications [6, 24]. In experimental studies, the effect of myocardial preconditioning is tremendous. Disappointingly, in clinical studies, the effect of myocardial preconditioning is less prevalent or even absent [2].

Comorbidities and age may, at least in part, explain these limited benefits of preconditioning in clinical studies. Preconditioning effects have been shown to be mitigated in the presence of different comorbidities such as artherosclerosis [10, 25]. Arteriosclerosis can impair the endothelial NOS function, and in eNOS-deficient mice, preconditioning is absent [11, 26]. However, reports of preconditioning in atherosclerotic animals are inconsistent [12, 15]. The aim of this study was to use an in vivo murine atherosclerosis model to investigate if remote and anesthetic-induced preconditioning is impaired in these animals. Surprisingly, in our experiments, preconditioning was preserved in LDL receptor-deficient mice with dietary-induced arteriosclerosis.

In our study, we used an established animal model of atherosclerosis and fed LDLR-deficient mice a high-fat diet [27]. The development of atherosclerosis was verified by histological examinations of aortic cross sections that confirmed arteriosclerotic alterations of the aortic intima-media of the mice. Whereas arteriosclerosis affects all blood vessels as a systemic disease [28, 29], atherosclerosis does not progress linearly and the manifestation pattern of the vessels is inhomogeneous. The development of arteriosclerosis is a complex process, especially in the initial phase. Histological changes in the aorta can be detected early, while changes in the small vessels might occur later [30, 31]. Since we did not perform a histological examination of the coronary arteries, we cannot exclude that atherosclerosis and endothelial dysfunction of the coronary arteries were in their very early stages in our experiments. Interestingly, another study conducted on isolated mouse hearts with severe atherosclerosis still reported preserved preconditioning [15]. Ischemic preconditioning is probably mediated in a paracrine manner, whereas remote preconditioning can only be mediated by systemic messengers. As the exact mechanisms are still not fully elucidated, it remains unclear if the stage of atherosclerosis in the coronary arteries of mice is of any relevance for the preserved preconditioning effects we report.

In addition to morphological investigations, we conducted experiments to examine the functional amount of atherosclerosis and tested the relaxation of small vessels (SMA sections) using a myograph. Even if there was some residual eNOS-mediated vessel relaxation in atherosclerotic LDLR-/- mice, the responsiveness of the LDLR-/- mouse vessels to relaxation decreased overall. These data provide evidence that atherosclerosis with endothelial dysfunction developed on a systemic level in our experimental model. However, since preconditioning is mediated at least in part by endothelial NO synthase [11], we had expected our model to show at least a reduction in infarct size through preconditioning even with residual eNOS activity. The completely preserved preconditioning effect cannot be conclusively explained by our findings. It is clear that robust cardioprotective effects independent of preconditioning were activated in the mice used in our experiments, since the myocardial infarct size was reduced in the high-fat fed LDLR-/- mice compared to WT mice under control conditions. The assumption that a high-fat diet as such in LDLR-/- mice leads to cardioprotection is supported by data from Girod et al., who reported that a longer hypercholesterolemic diet in LDLR-/- mice resulted in a reduction in IRI damage [32].

Surprisingly, preconditioning was not only preserved in atherosclerotic LDLR-/- mice, but it indeed added to the otherwise induced cardioprotection. A possible explanation could be the interaction of eNOS and glutathione. In their study, Girod et al. were able to demonstrate an increased glutathione content in the myocardium in LDLR-/- mice with a hypercholesterolemic diet [32]. Glutathione is a radical scavenger and thus plays an important role in ischemia reperfusion injury. If increased glutathione levels due to long-term hypercholesterolemia indeed mediate cardioprotective effects, this interaction with preconditioning has to be investigated in future studies.

## 5. Conclusion

Taken together, long-term high-fat atherogenic diet and preconditioning (APC/RIPC) seem to be an additive cardioprotection in LDLR-/- mice. A cross-species statement about factors influencing precondition ability does not seem to be possible.

## Figures and Tables

**Table 1:** The body weight of LDLR-/- mice fed a atherogenic diet was significantly higher compared to that of the WT mice.

	CON	APC	RIPC
WT	$\left.\begin{array}{c} 25.0\text{ g}\pm 0.4\text{ g}\\ 31.0\text{ g}\pm 1.1\text{ g} \end{array}\right\}*$	$\left.\begin{array}{c} 24.5\text{ g}\pm 0.6\text{ g}\\ 30.1\text{ g}\pm 0.6\text{ g} \end{array}\right\}*$	$\left.\begin{array}{c} 25.1\text{ g}\pm 0.3\text{ g}\\ 30.0\text{ g}\pm 0.5\text{ g} \end{array}\right\}*$
LDLR-/-			

^∗^ *p* < 0.05.

**Table 2:** Hemodynamic parameters of WT and LDLR-/- mice with and without preconditioning.

Group	MEM15′ HR (min^−1^)	MEM15′ MAP (mmHg)	CAO HR (min^−1^)	COA MAP (mmHg)	REP60′ HR (min^−1^)	REP60′ MAP (mmHg)
CON WT	$\left.\begin{array}{c} 688\pm 23\\ 371\pm 15 \end{array}\right\}*$	$\left.\begin{array}{c} 39\pm 4\\ 48\pm 6 \end{array}\right\}*$	$\left.\begin{array}{c} 663\pm 26\\ 379\pm 15 \end{array}\right\}*$	$\left.\begin{array}{c} 33\pm 3\\ 44\pm 6 \end{array}\right\}*$	$\left.\begin{array}{c} 638\pm 18\\ 371\pm 10 \end{array}\right\}*$	$\left.\begin{array}{c} 32\pm 3\\ 46\pm 7 \end{array}\right\}*$
CON LDLR-/-						
SEVO WT	$\left.\begin{array}{c} 556\pm 98\\ 494\pm 18 \end{array}\right\}*$	$\left.\begin{array}{c} 34\pm 6\\ 41\pm 7 \end{array}\right\}*$	$\left.\begin{array}{c} 557\pm 100\\ 488\pm 25 \end{array}\right\}*$	$\left.\begin{array}{c} 31\pm 5\\ 41\pm 6 \end{array}\right\}*$	$\left.\begin{array}{c} 505\pm 100\\ 469\pm 23 \end{array}\right\}*$	$\left.\begin{array}{c} 38\pm 7\\ 33\pm 6 \end{array}\right\}*$
SEVO LDLR-/-						
RIPC WT	342 ± 8	$\left.\begin{array}{c} 55\pm 4\\ 63\pm 7 \end{array}\right\}*$	350 ± 5	45 ± 3	342 ± 8	49 ± 4
RIPC LDLR-/-	367 ± 6		358 ± 8	49 ± 7	350 ± 13	52 ± 8

^∗^ *p* < 0.05.

## Data Availability

The data of this study are available on request from the authors.
